# Molecular Analysis of IL-5 Receptor Subunit Alpha as a Possible Pharmacogenetic Biomarker in Asthma

**DOI:** 10.3389/fmed.2020.624576

**Published:** 2021-02-11

**Authors:** Sandra Elena-Pérez, David Hansoe Heredero-Jung, Asunción García-Sánchez, Miguel Estravís, Maria J. Martin, Jacinto Ramos-González, Juan Carlos Triviño, María Isidoro-García, Catalina Sanz, Ignacio Dávila

**Affiliations:** ^1^Department of Clinical Biochemistry, University Hospital of Salamanca, Salamanca, Spain; ^2^Allergic Disease Research Group IIMD-01, Institute for Biomedical Research of Salamanca, Salamanca, Spain; ^3^Department of Biomedical Sciences and Diagnostics, University of Salamanca, Salamanca, Spain; ^4^Network for Cooperative Research in Health - RETICS ARADyAL, Carlos III Health Institute, Madrid, Spain; ^5^Department of Pneumology, University Hospital of Salamanca, Salamanca, Spain; ^6^Sistemas Genómicos, Paterna, Spain; ^7^Department of Medicine, University of Salamanca, Salamanca, Spain; ^8^Department of Microbiology and Genetics, University of Salamanca, Salamanca, Spain; ^9^Department of Allergy, University Hospital of Salamanca, Salamanca, Spain

**Keywords:** asthma, pharmacogenetic biomarker, transcriptomic, *IL5RA*, benralizumab, precision medicine

## Abstract

**Background:** Asthma is a heterogeneous syndrome with a broad clinical spectrum and high drug response variability. The inflammatory response in asthma involves multiple effector cells and mediator molecules. Based on asthma immunopathogenesis, precision medicine can be a promising strategy for identifying biomarkers. Biologic therapies acting on the IL-5/IL-5 receptor axis have been developed. IL-5 promotes proliferation, differentiation and activation of eosinophils by binding to the IL-5 receptor, located on the surface of eosinophils and basophils. This study aimed to investigate the expression of *IL5RA* in patients with several types of asthma and its expression after treatment with benralizumab, a biologic directed against IL-5 receptor subunit alpha.

**Methods:** Sixty peripheral blood samples, 30 from healthy controls and 30 from asthmatic patients, were selected for a transcriptomic RNAseq study. Differential expression analysis was performed by statistical assessment of fold changes and *P*-values. A validation study of *IL5RA* expression was developed using qPCR in 100 controls and 187 asthmatic patients. The effect of benralizumab on *IL5RA* expression was evaluated in five patients by comparing expression levels between pretreatment and after 3 months of treatment. The *IL5RA* mRNA levels were normalized to *GAPDH* and *TBP* expression values for each sample. Calculations were made by the comparative ΔΔCt method. All procedures followed the MIQE guidelines.

**Results:***IL5RA* was one of the most differentially overexpressed coding transcripts in the peripheral blood of asthmatic patients (*P* = 8.63E-08 and fold change of 2.22). In the qPCR validation study, *IL5RA* expression levels were significantly higher in asthmatic patients than in controls (*P* < 0.001). Significant expression differences were present in different asthmatic types. In the biological drug study, patients treated with benralizumab showed a significant decrease in *IL5RA* expression and blood eosinophil counts. A notable improvement in ACT and lung function was also observed in these patients.

**Conclusions:** These results indicate that *IL5RA* is overexpressed in patients with different types of asthma. It could help identify which asthmatic patients will respond more efficiently to benralizumab, moving toward a more personalized asthma management. Although further studies are required, *IL5RA* could play a role as a biomarker and pharmacogenetic factor in asthma.

## Introduction

Asthma is a chronic inflammatory disease of the airways affecting more than 300 million people worldwide, and its prevalence is increasing, becoming a health and economic problem (1). It is defined by variable expiratory airflow limitation and respiratory symptoms such as wheeze, cough and shortness of breath, which vary in frequency and intensity (2). Asthma is recognized as a heterogeneous syndrome with different underlying disease processes, determined by complex interactions between genetic and environmental factors. This variety of interactions results in different clinical presentations, phenotypes and response to treatment (3).

Despite the broad clinical spectrum of asthma, the presence of inflammation of the airway is a common pathologic feature and the primary treatment target. Nevertheless, the relationship between the intensity of inflammation and the severity of asthma has not been consistently established (4). The characteristic inflammatory pattern in most asthmatic patients includes an increase in type 2 helper T (Th2) lymphocytes, eosinophils, basophils, mast cells and type 2 innate lymphoid cells (ILC2). These effector cells release numerous mediating molecules that cause disease symptoms (5). Type 2 inflammation is characterized by an increase in type 2 cytokines, particularly IL-4, IL-5, IL-9, and IL-13, involved in eosinophil activation and immunoglobulin E production (6).

Asthma comprises different phenotypes with similar clinical manifestations but probably involving different underlying mechanisms (7). These phenotypes have been characterized based on the age of onset of disease, clinical presentation, severity and presence of other disorders such as atopy and eosinophilia (8–10). Eosinophilic asthma is the best-studied inflammatory phenotype and is characterized by elevated eosinophils in peripheral blood and sputum. Patients in whom eosinophilic inflammation persists despite treatment with high doses of corticosteroids are often associated with more severe asthma and a higher risk of exacerbations (11, 12). Therefore, phenotype characterization in patients with severe uncontrolled or poorly controlled asthma could help in guiding specific treatments (13).

Asthma management aims to achieve and maintain control of the disease and reduce the risk of exacerbations. Understanding the underlying pathophysiologic mechanisms is necessary for stratifying patients toward individualized therapy. The implementation of precision medicine requires identifying specific biomarkers easily measurable in biological fluids, which can help in evaluating treatment effectiveness (14, 15). In this context, new biologic therapies are being developed targeting cytokines and their receptors. IL-5 plays a crucial role in eosinophilic asthma pathophysiology and has been proposed as a novel therapeutic target. This cytokine is involved in the proliferation, differentiation, survival and activation of eosinophils by binding to the IL-5 receptor, located on the surface of eosinophils and basophils (16, 17). The IL-5 receptor is a heterodimer comprising one alpha subunit (IL-5RA) and one beta subunit, also found in both IL-3 and GM-CSF receptors (18).

In recent years, new drugs based on monoclonal antibodies have been developed against the action of IL-5 in eosinophilic-mediated inflammation. Benralizumab is a humanized IgG1κ monoclonal antibody that binds to IL-5RA via its Fab domain with high affinity and specificity, blocking IL-5 signaling (19). Besides, this antibody can bind through its afucosylated Fc domain to the Fcγ receptor IIIa, expressed on the surface of natural killer cells, macrophages and neutrophils, thus inducing antibody-directed cell-mediated cytotoxicity of eosinophils and basophils. As a result, the administration of benralizumab results in a dramatic depletion of eosinophils counts in blood, sputum, airway mucosa and bone marrow (20). In this context, benralizumab has proved effectivity in treating patients with severe eosinophilic asthma, improving lung function and asthma control and reducing the rate of exacerbations (21).

The present study aims to investigate the expression of *IL5RA* in patients with different types of asthma and its role as a possible biomarker of response to treatment with benralizumab.

## Materials and Methods

### Study Population

The study involved 347 unrelated Caucasian individuals, 130 controls and 217 asthmatic patients, from the Allergy Department of the University Hospital of Salamanca. The study was approved by the Clinical Research Ethics Committee of the Institute for Biomedical Research of Salamanca (IBSAL) (PI 2020-02-433) and all participants signed a written informed consent. Controls had to fulfill the following criteria: (i) no symptoms or history of asthma, rhinitis or other pulmonary diseases; (ii) no symptoms or history of allergic diseases; (iii) negative skin prick tests with a battery of locally adapted common aeroallergens; (iv) absence of family history of asthma, rhinitis or atopy; and (v) age >16 years old. Asthmatic patients were recruited if they met all the following criteria: (i) at least two symptoms consistent with asthma (cough, wheeze and dyspnea); (ii) either a positive bronchodilator or methacholine test; and (iii) absence of other pulmonary disorders; and (iv) age >16 years old.

Lung function was measured by spirometry according to the American Thoracic Society (ATS) criteria (22). Asthma severity was established following the Spanish Guide for the Management of Asthma (GEMA) guidelines (7) and severe asthma was diagnosed according to the ERS/ATS criteria (23). Skin prick tests were performed with a battery of common aeroallergens (24), according to The European Academy of Allergy and Clinical Immunology (EAACI) recommendations (25). Skin tests were considered positive if there was at least one wheal reaction of >3 mm of diameter. Patients were considered atopic if they had a positive skin prick test to at least one allergen. Patients were considered monosensitized if they had a positive skin prick test result to only one group of aeroallergens (pollens, mites, molds or animal dander) and polysensitized if they had positive skin tests for two or more groups. Early-onset asthma was defined as the presence of asthma symptoms that appeared before 18 years, and late-onset asthma was defined as the presence after the age of 18 years (26). Asthmatic patients were classified into two subgroups, eosinophilic and non-eosinophilic asthma, according to the number of eosinophils (cut-off point of 150 cells per μl) (2). Blood cell counts were determined on the XN-1000 hematology analyzer (Sysmex Corporation, Kobe, Japan) and total serum IgE levels were measured using a fluoroenzyme immunoassay (Thermo Fisher Scientific, Waltham, MA, USA).

### Transcriptomic RNAseq Study

A total of 60 peripheral blood samples, 30 from healthy controls and 30 from patients with pollen allergic asthma, were selected for a transcriptomic RNAseq study. Total RNA extraction was performed using the Ambion RiboPure^TM^-Blood kit (Thermo Fisher Scientific, Waltham, MA, USA). After Ambion DNAse I treatment (Thermo Fisher Scientific, Waltham, MA, USA), RNA was purified and concentrated with the RNeasy MinElute Cleanup Kit (Qiagen, Hilden, Germany). All purification protocols were performed with the modifications indicated by the manufacturers. RNA was quantified by Nanodrop 1000 spectrophotometer (Thermo Fisher Scientific, Waltham, MA, USA). The RNA integrity number (RIN) algorithm was used to determine RNA quality on the Agilent 2100 Bioanalyzer using the Eukaryote Total RNA Nano kit (Agilent Technologies, Waldbronn, Germany). RNA samples with a RIN value above 8 were used. Globin transcripts and ribosomal RNA were removed, and RNA was cleaved to prepare RNA strand-specific libraries. Finally, the generated libraries were sequenced on the Illumina HiSeq 2500 platform (Illumina, San Diego, CA, USA).

### Bioinformatic Analysis

In the bioinformatic analysis of the transcriptomic data, the FastQC software (27) was used to analyze the quality of raw reads. Sequencing reads were mapped on the human reference genome (GRCh38) using the TopHat2 software (28). The low-quality readings were removed with Picard Tools (29) and the unmapped and non-properly paired reads were re-mapped using the BWA-MEM algorithm (30). Gene and isoform prediction were estimated using the Cufflinks method (31). The HTSeq software (v.0.6.0) (32) was used to calculate gene expression levels. Differential expression analysis was performed by DESeq2 package (33) and only the transcripts with a fold change value ≥1.5 or ≤ −1.5 and a FDR-adjusted *P*-value <0.05 were considered as differentially expressed genes. Potential interactions between selected proteins were examined by cluster analysis using the STRING software (34), a database which include functional and physical associations between known and predicted proteins.

### qPCR Validation Assays

For the validation of the transcriptomic gene expression data, 287 peripheral blood samples were selected including 100 samples from controls and 187 from asthmatic patients. Total RNA was isolated using the RiboPure-Blood kit (Ambion, Thermo Fisher Scientific, Waltham, MA, USA). DNAse treatment was performed using Ambion DNAse I (Thermo Fisher Scientific, Waltham, MA, USA). Concentrations and RNA quality ratios were determined in a Nanodrop 1000 spectrophotometer (Thermo Fisher Scientific, Waltham, MA, USA). cDNA was generated from 500 ng of total RNA using Superscript III First-Strand Synthesis System for RT-PCR (Invitrogen, Thermo Fisher Scientific, Waltham, MA, USA), in a final volume of 20 μl. Conditions for PCR included a single cycle and incubation periods of 65°C for 5 min, 25°C for 10 min, 50°C for 50 min, 85°C for 5 min, and 37°C for 20 min.

qPCR reactions were performed in a LightCycler480 system (Roche Applied Science, Indianapolis, IN, USA). *IL5RA* primers were designed using Primer 3.0 (35) and the Beacon Designer (36) software. *GAPDH* and *TBP* reference gene primers were chosen from The Real Time ready Human Reference GenePanel (Roche Applied Science, Indianapolis, IN, USA). The sequence of the primers used are shown in Table 1. Primers efficacies were analyzed by amplifying serial dilutions of cDNA sample of known concentration and according to the following equation: E = (10^−1/*slope*^ − 1) × 100. All efficiencies ranged from 90 to 110%. The reaction mixture in each well-contained a final volume of 15 μl based on 7.5 μl of Master Mix SYBR Green I (Roche Applied Science, Indianapolis, IN, USA), 10 μM of each primers and 20 ng of cDNA. All reactions were performed in triplicate. In each experiment, non-template controls and calibrator were included. The PCR conditions included 10 min at 95°C followed by 45 cycles of 10 s at 95°C for denaturation, 10 s at 60°C for annealing and 10 s at 72°C for polymerization. Finally, melting curve analyses were carried out to verify the specificity of the qPCR products. *IL5RA* mRNA levels were normalized to *GAPDH* and *TBP* expression levels using the formula 2^−ΔΔCt^ by the comparative ΔΔCt method (37). All procedures followed the Minimum Information for Publication of Quantitative Real-Time PCR Experiment (MIQE) guidelines (38).

**Table 1:** Sequences of primers used in the qPCR assay.

	**Primer**	**Sequence 5^**′**^ → 3^**′**^**
*IL5RA*	Forward	TGAAAGAGTGAAGAACCGCC
	Reverse	CCTGGCCTGAGAAATGCG
*GAPDH*	Forward	CTCTGCTCCTCCTGTTCGAC
	Reverse	ACGACCAAATCCGTTGACTC
*TBP*	Forward	GAACATCATGGATCAGAACAACA
	Reverse	ATAGGGATTCCGGGAGTCAT

### Pharmacogenetic Study

A proof-of-concept study of the *IL5RA* expression in peripheral blood before and after 3 months of treatment with benralizumab was performed in five severe eosinophilic asthmatic patients. Benralizumab was administered at a dose of 30 mg by subcutaneous injection once every 4 weeks. Asthma control test (ACT), fractional exhaled nitric oxide (FeNO) and lung function parameters were performed before and after 3 months of treatment. Patients were considered responders in the ACT score if they achieved a score of 25 or an increase of 3 or more points after 3 months of treatment. Also, they were considered FEV1 responders if they achieved a FEV1.0 ≥ 200 ml or FEV1.0 ≥ 12% after 3 months of treatment. As only three doses were evaluated, exacerbations were not considered. Blood samples were collected and lung function tests were performed at each time point. All patients provided their informed consent to receive benralizumab therapy.

### Statistical Analysis

Descriptive analysis was carried out using central (mean and median) and dispersion tendency (standard deviation and interquartile range) measurements, followed by bivariate and multivariate analysis. The normality distribution was assessed by Kolmogorov-Smirnov test and the homoscedasticity was also tested before applying statistical tests. Continuous variables were evaluated using either ANOVA or Kruskal-Wallis test. Statistical significance was assessed by Wilcoxon's test for changes before and after treatment. A *P*-value <0.05 was considered statistically significant. All statistical analyses were performed using SPSS Statistics version 21 (IBM, Armonk, NY, USA). Graphs were plot using GraphPad Prism version 6 (San Diego, CA, USA).

## Results

### Study Population

The phenotypic characteristics of the studied subjects of the RNAseq study and the validation analysis are shown in Table 2. In both assays, control individuals were older to permit a more extended period for asthma to have appeared. Thus, age was significantly higher in the control group than in patients (*P* < 0.001), except in the case of non-allergic and late-onset asthma groups, in which the disease had begun at older ages. According to the inclusion criteria, total IgE levels were significantly higher in all patient groups than in controls (*P* ≤ 0.001). Moderate persistent asthma was the most common type in both the RNAseq study and the validation analysis (43.3 and 45.7%, respectively), followed by intermittent asthma (33.3 and 26.3%, respectively). The most common aeroallergen sensitization in the validation analysis patients was pollen, followed by animal dander. No patient was receiving oral corticosteroids.

**Table 2:** Characteristics of the study population in the RNAseq study and the validation analyses.

	***N***	**Sex (% Female)**	**Age (Mean ± SD)**	**FeNO, ppb (Mean ± SD)**	**IgE, kU/l (Median ± IQR)**
**RNAseq study**					
Controls	30	46.7	57 ± 17	-	46.7 ± 87.8
Asthmatic patients	30	56.7	30 ± 13	-	179.0 ± 239.0
**Validation analysis**					
Controls	100	66.0	57 ± 17	-	28.8 ± 57.7
Asthmatic patients	187	55.1	45 ± 19	44.8 ± 48.3	174.0 ± 398.2
Non-allergic asthma (NAA)	76	60.5	58 ± 15	38.1 ± 40.6	77.0 ± 118.8
NAA without NP	33	72.7	56 ± 16	24.8 ± 19.3	37.9 ± 103.5
NAA with NP	43	51.2	61 ± 14	52.2 ± 51.7	83.0 ± 258.7
Allergic asthma (AA)	111	51.4	35 ± 16	49.8 ± 53.0	312.5 ± 472.3
AA without NP	82	57.3	31 ± 14	39.8 ± 29.5	312.5 ± 445.5
AA with NP	29	34.5	46 ± 17	79.6 ± 88.7	307.0 ± 747.0
Early-onset asthma	76	61.8	31 ± 17	42.5 ± 43.1	271.0 ± 402.1
Late-onset asthma	111	50.5	54 ± 16	46.5 ± 51.9	126.0 ± 406.5
Non-eosinophilic asthma	32^a^	59.4	44 ± 17	26.4 ± 18.2	109.0 ± 260.5
Eosinophilic asthma	120^a^	53.3	46 ± 20	49.7 ± 54.5	196.0 ± 405.4

*SD, standard deviation; IQR, interquartile range*.

a *Blood eosinophil counts were not available for all non-eosinophilic and eosinophilic asthma patients*.

### Transcriptomic RNAseq Study

Significant differences between control and asthmatic patients were observed in the transcriptomic assay (*P* < 0.05). Table 3 shows the top 26 most differentially expressed transcripts between controls and patients with allergic asthma, according to *P*-value and fold change. The main biological roles of these genes are described in Table 4, which highlights the biological processes related to the immune system. Among these genes, *IL5RA* attracted our attention as a putative asthma biomarker because it was the best positioned when considering both fold change and *P*-value data, and because of its role in different immune processes.

**Table 3:** The 26 protein-coding transcripts most differentially expressed (*P* < 0.025) between the group of controls and patients with allergic asthma, listed by their fold change value.

**Ensemble ID**	**External ID gene**	**Fold change**	***P*-value**
**Up-regulated expression**			
ENSG00000161905	*ALOX15*	2.45	3.91E-05
ENSG00000091181	*IL5RA*	2.22	8.63E-08
ENSG00000103056	*SMPD3*	2.16	1.44E-07
ENSG00000105205	*CLC*	2.04	4.63E-06
ENSG00000183134	*PTGDR2*	1.99	2.64E-06
ENSG00000134489	*HRH4*	1.93	7.21E-07
ENSG00000152207	*CYSLTR2*	1.84	1.15E-09
ENSG00000171659	*GPR34*	1.83	1.27E-08
ENSG00000143297	*FCRL5*	1.75	3.18E-05
ENSG00000255587	*RAB44*	1.74	8.10E-06
ENSG00000132465	*JCHAIN*	1.70	0.007
ENSG00000276231	*PIK3R6*	1.67	9.12E-06
ENSG00000131203	*IDO1*	1.67	0.014
**Down-regulated expression**			
ENSG00000118113	*MMP8*	−2.75	3.17E-04
ENSG00000012223	*LTF*	−2.35	4.79E-04
ENSG00000124469	*CEACAM8*	−2.19	2.47E-03
ENSG00000123689	*G0S2*	−2.02	4.95E-03
ENSG00000118520	*ARG1*	−2.00	4.51E-05
ENSG00000168209	*DDIT4*	−1.91	4.11E-04
ENSG00000179094	*PER1*	−1.86	6.80E-04
ENSG00000096006	*CRISP3*	−1.78	0.002
ENSG00000005961	*ITGA2B*	−1.75	6.90E-04
ENSG00000179869	*ABCA13*	−1.73	0.001
ENSG00000100985	*MMP9*	−1.69	0.001
ENSG00000124102	*PI3*	−1.65	7.32E-04
ENSG00000122025	*FLT3*	−1.64	3.66E-05

**Table 4:** Gene Ontology term enrichment analysis of the more differentially expressed genes.

**Term ID**	**Biological process**	**FDR**	**Genes**
**Immune system**			
GO:0002376	Immune system process	1.74E-07	*ABCA13, ARG1, CEACAM8, CRISP3, CYSLTR2, DDIT4, FLT3, IDO1, IL5RA, LTF, MMP8, MMP9, PI3, PTGDR2, RAB44, SMPD3*.
GO:0006955	Immune response	5.94E-06	*ABCA13, ARG1, CEACAM8, CRISP3, CYSLTR2, IL5RA, LTF, MMP8, MMP9, PI3, PTGDR2, RAB44*.
GO:0043312	Neutrophil degranulation	7.28E-05	*ABCA13, ARG1, CEACAM8, CRISP3, LTF, MMP8, MMP9, RAB44*.
**Cellular process**			
GO:0001775	Cell activation	1.32E-06	*ABCA13, ARG1, CEACAM8, CRISP3, FLT3, ITGA2B, LTF, MMP8, MMP9, PIK3R6, RAB44*.
GO:0019221	Cytokine-mediated signaling pathway	8.10E-03	*ALOX15, FLT3, IL5RA, MMP9*.
GO:0007166	Cell surface receptor signaling pathway	0.017	*ALOX15, DDIT4, FLT3, G0S2, GPR34, IL5RA, MMP9*.
GO:0007165	Signal transduction	0.012	*ALOX15, CYSLTR2, DDIT4, FLT3, G0S2, GPR34, HRH4, IL5RA, MMP9, PIK3R6, PTGDR2*.
**Cellular response**			
GO:0050896	Response to stimulus	2.52E-06	*ABCA13, ALOX15, ARG1, CEACAM8, CRISP3, CYSLTR2, DDIT4, FLT3, G0S2, GPR34, HRH4, IDO1, IL5RA, ITGA2B, LTF, MMP8, MMP9, PER1, PI3, PIK3R6, PTGDR2, RAB44*.
GO:0006952	Defense response	9.28E-05	*ALOX15, ARG1, CRISP3, DDIT4, HRH4, IDO1, IL5RA, LTF*.
GO:0006950	Response to stress	3.00E-04	*ALOX15, ARG1, CRISP3, DDIT4, HRH4, IDO1, IL5RA, ITGA2B, LTF, MMP9, PIK3R6*.
GO:0006954	Inflammatory response	2.50E-03	*ALOX15, HRH4, IDO1, IL5RA*.
GO:0071345	Cellular response to cytokine stimulus	9.30E-03	*ALOX15, ARG1, FLT3, IL5RA, MMP9*.
**Biological regulation**			
GO:0048583	Regulation of response to stimulus	6.84E-05	*ALOX15, ARG1, CLC, CYSLTR2, DDIT4, FLT3, G0S2, HRH4, IDO1, LTF, MMP9, PER1, PIK3R6, PTGDR2*.
GO:0009966	Regulation of signal transduction	6.84E-05	*ALOX15, ARG1, CYSLTR2, DDIT4, FLT3, G0S2, HRH4, LTF, MMP9, PER1, PIK3R6 PTGDR2*.
GO:0051239	Regulation of multicellular organismal process	2.30E-04	*ARG1, CLC, CYSLTR2, IDO1, IL5RA, ITGA2B, LTF, MMP9, PER1, PIK3R6, PTGDR2*.
GO:0001817	Regulation of cytokine production	6.20E-03	*ARG1, CLC, IDO1, IL5RA, LTF, PER1*.
GO:0050794	Regulation of cellular process	0.018	*ALOX15, ARG1, CLC, CRY2, CYSLTR2, DDIT4, FLT3, G0S2, GPR34, HRH4, IDO1, IL5RA, ITGA2B, LTF, MMP9, PER1, PI3, PIK3R6, PTGDR2, SMPD3*.

*FDR, false discovery rate*.

**Figure 1:**
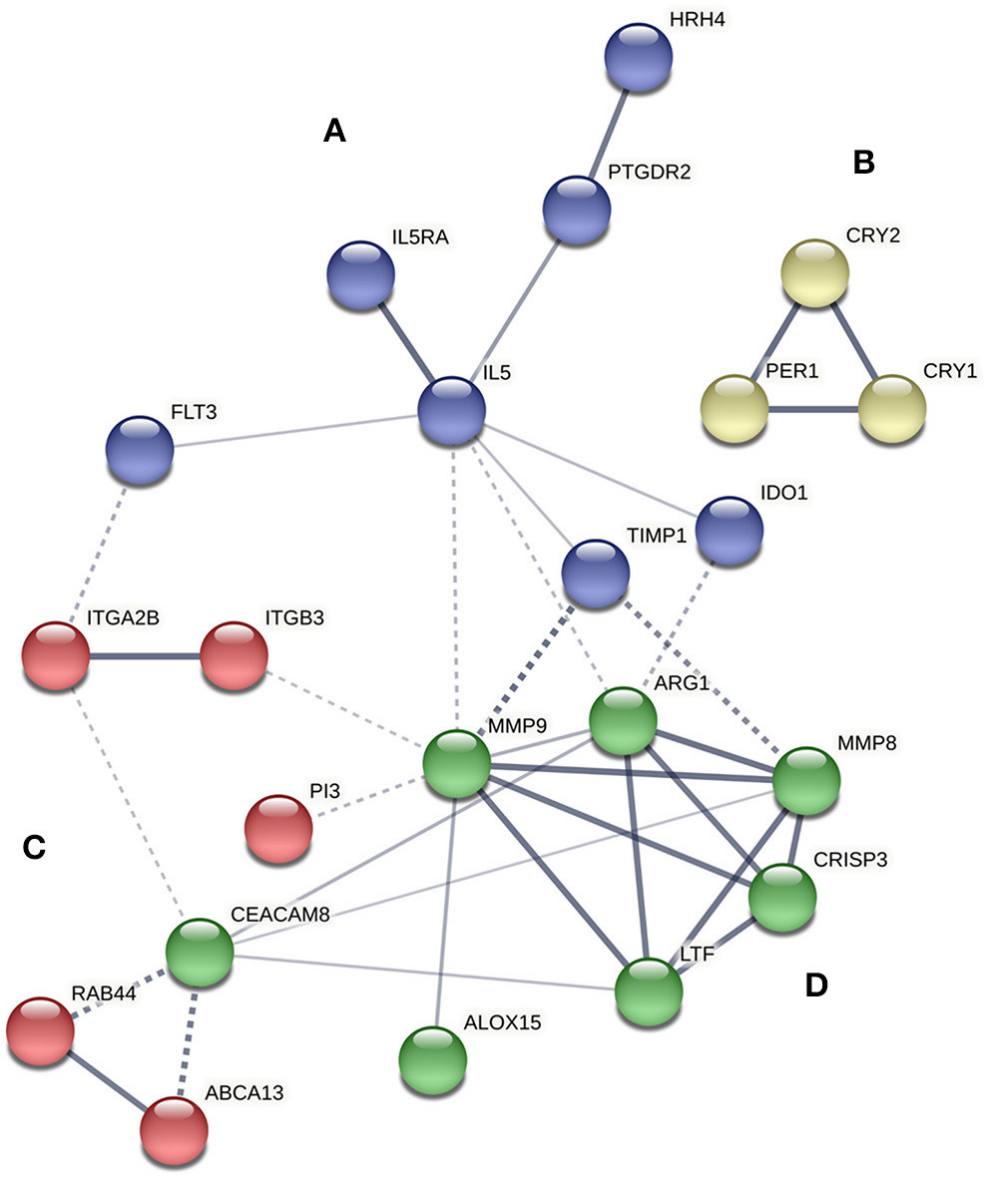
Protein interaction network of proteins encoded by differentially expressed genes between controls and asthmatic patients using STRING software. The strength of data support is indicated by line thickness. The four main clusters are shown in blue **(A)**, yellow **(B)**, red **(C)**, and green **(D)**.

A protein-protein interaction network analysis was performed with the selected transcripts to analyze the interactions among them. Four main clusters were obtained, referred to as clusters A, B, C, and D (Figure 1). *IL5RA* was found in cluster A, which included seven genes: *IL5RA, IL5, FLT3* (receptor-type tyrosine-protein kinase), *PTGDR2* (prostaglandin D2 receptor 2), *HRH4* (histamine H4 receptor), *TIMP1* (metalloproteinase inhibitor 1) and *IDO1* (indoleamine 2,3-dioxygenase 1). Regarding the role of *IL5RA* in biological processes, the most significant terms were “immune system process” (FDR 1.74E-07) and “response to stimulus” (FDR 2.52E-06) (Table 4), as well as in Reactome pathways, such as “signaling by interleukins” and “RAF/MAP kinase cascade.”

### qPCR Validation Assays

A validation qPCR analysis was performed to confirm the differences observed in the RNAseq study. Patients were classified according to the presence of atopy and nasal polyposis (NP), the severity of asthma and the age of onset of asthma. As summarized in Table 5, asthmatic patients had significantly higher levels of peripheral blood eosinophil counts and *IL5RA* expression than controls (*P* < 0.001). That also occurred in all subgroups except for non-eosinophilic asthma (*P* = 0.707). Interestingly, the lower increase in *IL5RA* expression levels was observed in patients with non-allergic asthma (NAA) without NP (10.3 ± 11.2; *P* = 0.037). Significant differences were observed when comparing these patients with the subgroup of patients who had NAA with NP (*P* = 0.047). These significant differences were not observed among the other subgroups, although *IL5RA* expression levels were significantly higher in eosinophilic asthma than in non-eosinophilic asthma (*P* < 0.001). Also, *IL5RA* expression levels were slightly higher in monosensitized patients to pollens.

**Table 5:** Blood eosinophil counts and *IL5RA* expression levels according to asthma diagnosis, sensitization and severity.

	***N***	**Eosinophils/μl^a^ (Mean ± SD)**	***IL5RA*, relative expression (Mean ± SD)**	***P*-value^b^**	***P*-value^c^**
Controls	100	127.8 ± 89.4	7.1 ± 6.3		
Asthmatic patients	187	380.4 ± 331.0	15.5 ± 15.4	<0.001	
Non-allergic asthma (NAA)	76	373.4 ± 367.1	13.9 ± 13.7	<0.001	
NAA without NP	33	223.7 ± 169.4	10.3 ± 11.2	0.037	0.047
NAA with NP	43	510.0 ± 441.6	16.6 ± 14.9	<0.001	
Allergic asthma (AA)	111	385.6 ± 303.3	16.6 ± 16.4	<0.001	
AA without NP	82	308.4 ± 222.9	16.5 ± 17.0	<0.001	0.982
AA with NP	29	577.1 ± 386.9	16.6 ± 15.0	<0.001	
Monosensitized to pollens	22	478.7 ± 301.6	20.2 ± 17.3	<0.001	
Monosensitized to animal dander	6	300.0 ± 205.2	21.4 ± 29.2	0.050	
Monosensitized to mites	10	411.1 ± 513.4	14.5 ± 14.5	0.064	
Polysensitized	73	358.8 ± 266.4	15.2 ± 14.7	<0.001	
Intermittent asthma	49	290.8 ± 199.3	12.0 ± 13.7	0.003	0.056
Mild persistent asthma	29	337.4 ± 192.7	17.7 ± 18.4	<0.001	
Moderate persistent asthma	85	380.5 ± 340.9	15.4 ± 13.4	<0.001	
Severe persistent asthma	23	574.0 ± 494.3	19.9 ± 20.3	<0.001	
Early-onset asthma	76	364.0 ± 307.4	14.5 ± 14.4	<0.001	
Late-onset asthma	111	391.1 ± 346.7	16.1 ± 16.0	<0.001	
Non-eosinophilic asthma	32	98.5 ± 66.1	6.6 ± 5.8	0.707	<0.001
Eosinophilic asthma	120	455.6 ± 332.9	18.2 ± 15.4	<0.001	

*SD, standard deviation*.

a *All P-value results for blood eosinophil counts were significant (P < 0.05) among each patient group vs. controls, except for non-eosinophilic asthma (P = 0.082)*.

b *P-value obtained for the comparison of IL5RA expression levels from each patient group vs. controls*.

c *P-value obtained for comparison of IL5RA expression levels from NAA without NP vs. NAA with NP; AA without NP vs. AA with NP; intermittent asthma vs. severe persistent asthma; and non-eosinophilic asthma vs. eosinophilic asthma, respectively*.

In general, there was an association between asthma severity and increased levels of *IL5RA* expression (Table 5). Patients with intermittent asthma had the lowest expression levels of *IL5RA* (12.0 ± 13.7), while patients with severe asthma had the highest levels (19.9 ± 20.3; *P* = 0.056). In addition, there was a statistically significant association between asthma severity and the number of eosinophils (*P* = 0.015).

To discard a possible influence of anti-inflammatory treatments on the expression of *IL5RA*, patients receiving inhaled corticosteroids or allergen immunotherapy were compared with patients not receiving these treatments. One hundred thirty-nine patients were receiving therapy with inhaled corticosteroids; 54 patients were receiving allergen immunotherapy. No statistically significant differences in the *IL5RA* expression levels were observed between patients receiving corticosteroid treatment or allergen immunotherapy and patients not receiving these treatments.

**Figure 2:**
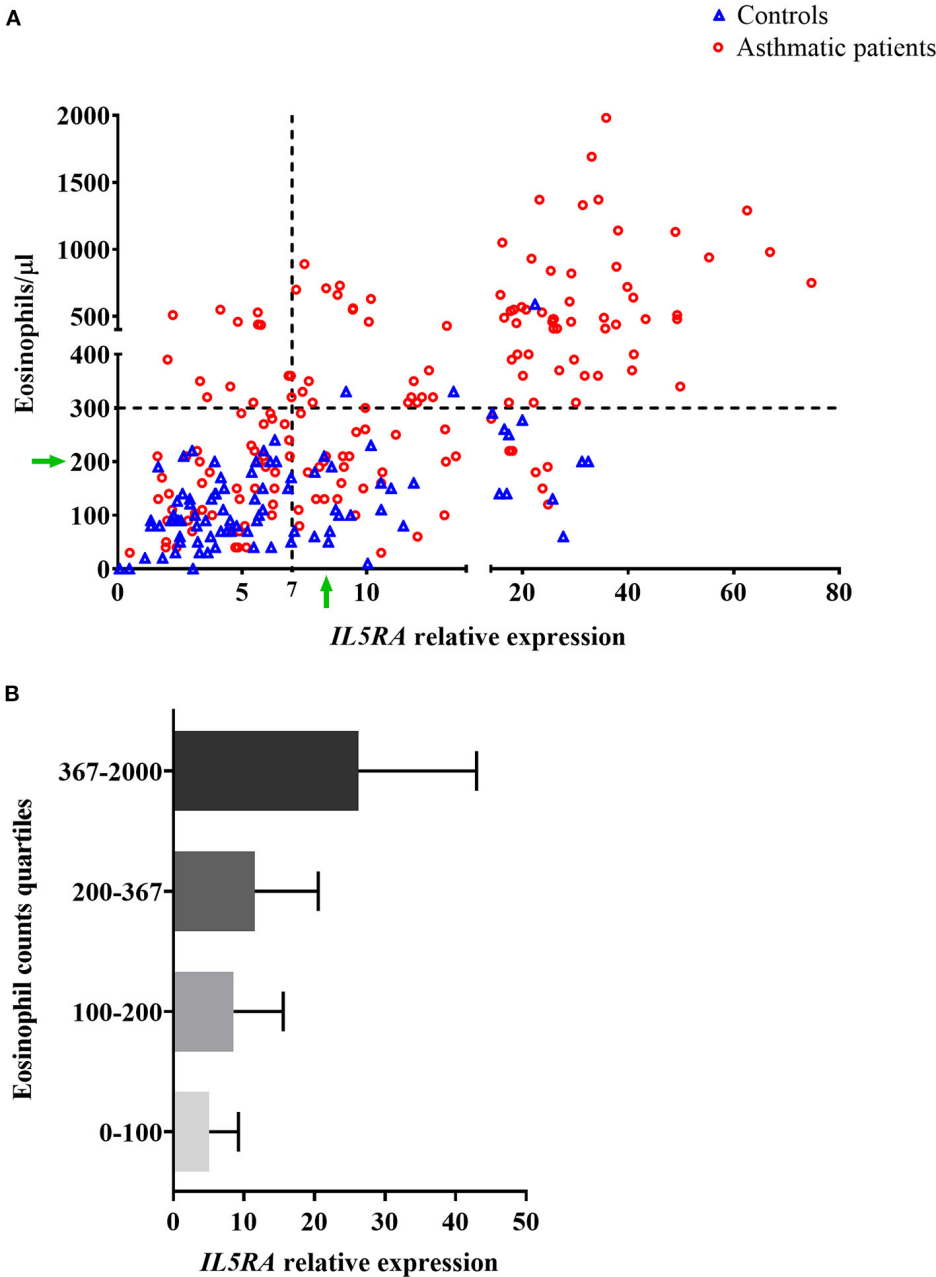
**(A)** Correlation between *IL5RA* expression levels and peripheral blood eosinophil counts of controls and asthmatic patients from the validation analysis. Four quadrants were obtained by dividing according to the normality values for eosinophil counts (300/μl) and *IL5RA* expression (7-fold). The green arrows indicate a constant value of eosinophils at which a wide range of expressed *IL5RA* values is observed, and vice versa. **(B)** *IL5RA* expression levels (mean ± SD) in eosinophil count quartiles.

**Figure 3:**
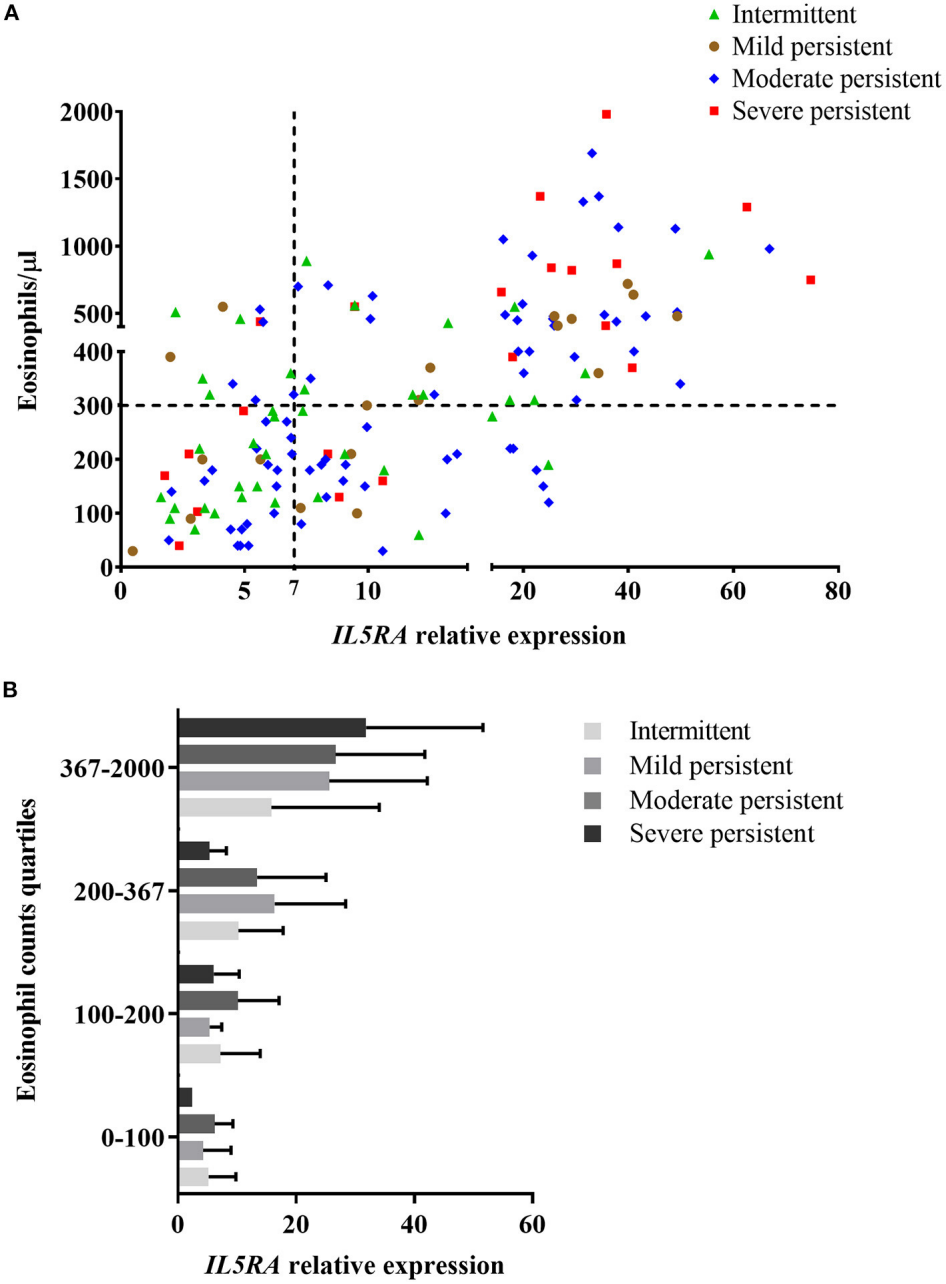
**(A)** Correlation between *IL5RA* expression levels and peripheral blood eosinophil counts according to asthma severity of patients from the validation analysis. Four quadrants were obtained by dividing according to the normality values for eosinophil counts (300/μl) and *IL5RA* expression (7-fold). **(B)** *IL5RA* expression levels (mean ± SD) in eosinophil count quartiles according to asthma severity.

The relationship between *IL5RA* expression levels and peripheral blood eosinophil counts was also analyzed, observing some correlation with a Pearson's correlation coefficient of 0.520 (*P* < 0.001). Remarkably, as shown in Figure 2A, there were patients with the same eosinophil counts and very different expression levels of *IL5RA*. In addition, an eosinophil count-dependent increase in both *IL5RA* expression levels and dispersion was observed when the eosinophil counts were divided into quartiles (Figure 2B). This fact was also observed according to asthma severity. The more severe the asthma was, the greater the *IL5RA* levels were (Figure 3).

### Pharmacogenetic Study

**Figure 4:**
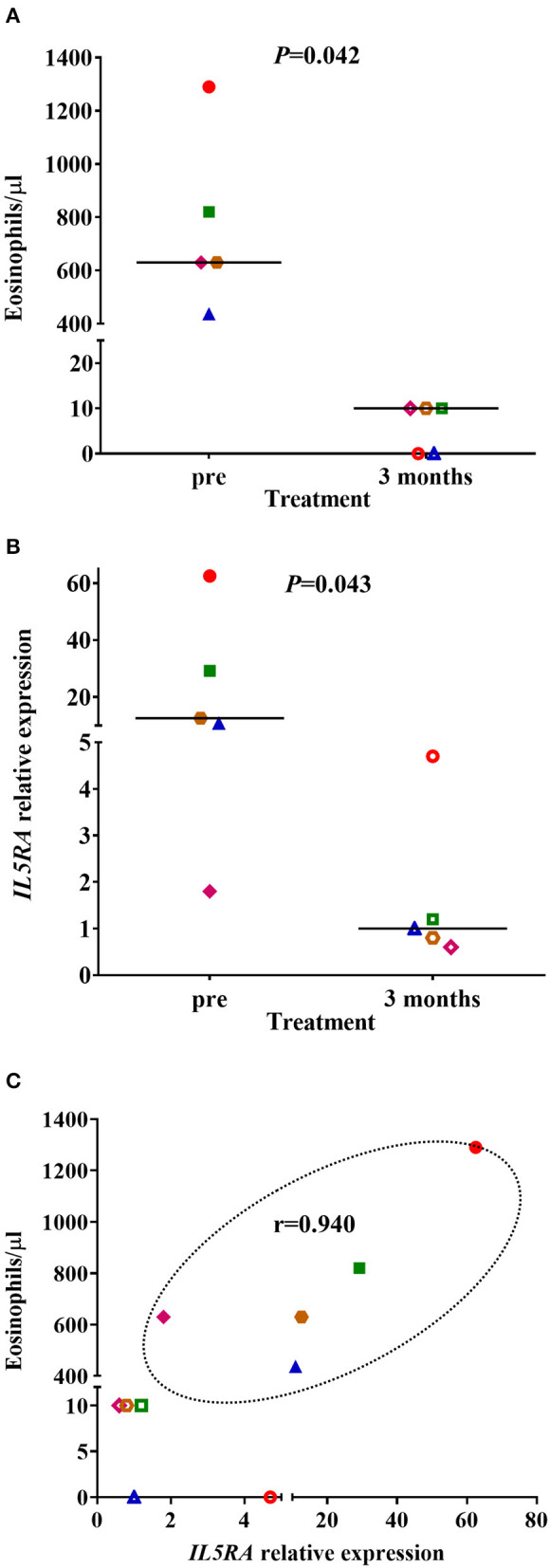
**(A)** Peripheral blood eosinophil counts and **(B)** *IL5RA* expression levels in pretreatment and after 3 months of benralizumab treatment. The median of each group is also shown. **(C)** Correlation between *IL5RA* expression levels and peripheral blood eosinophil counts at pretreatment and after 3 months of benralizumab treatment. Pearson's correlation coefficient in the pretreatment is also shown. Each symbol represents a patient (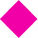 Patient 1; 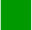 Patient 2; 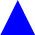 Patient 3; 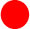 Patient 4; 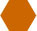 Patient 5). The filled symbols correspond to the pretreatment and the empty symbols to after 3 months of benralizumab treatment.

**Figure 5:**
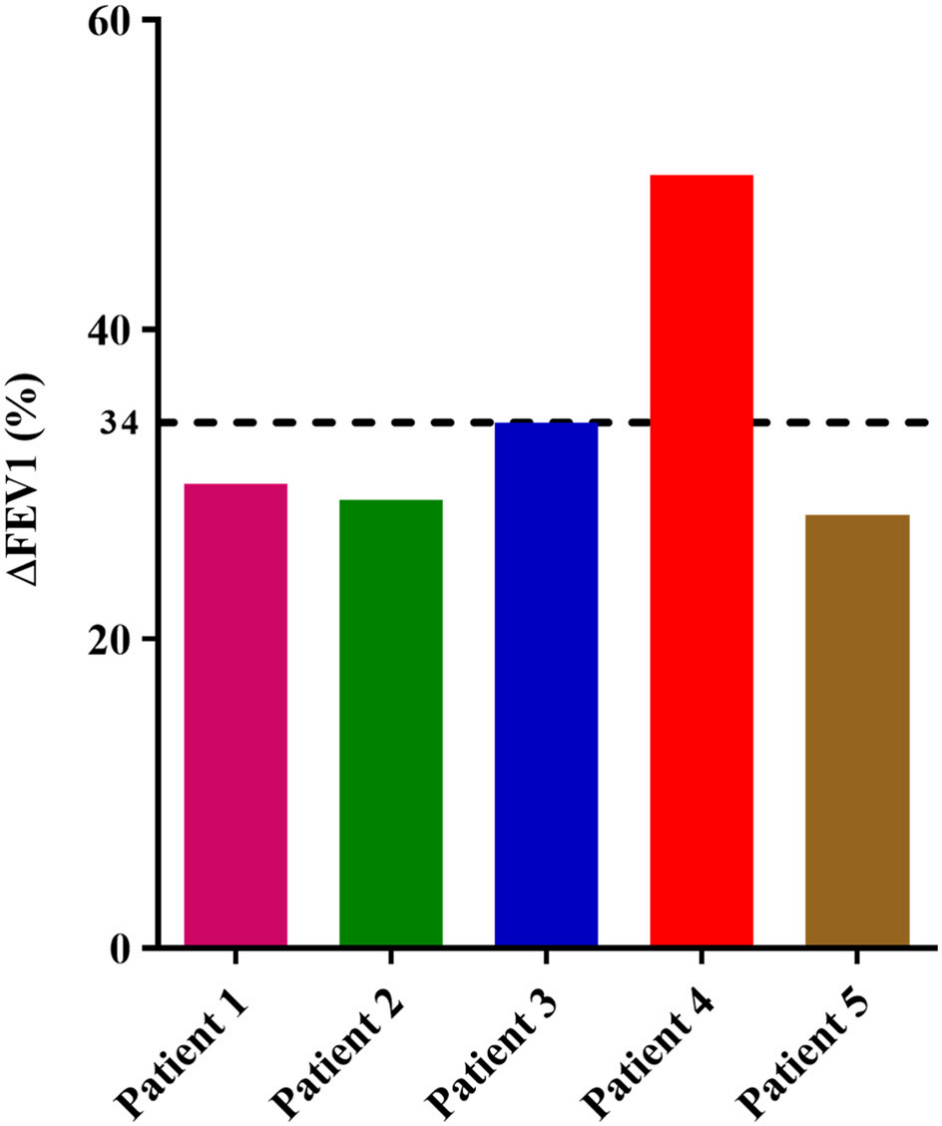
Percentage of FEV1 value variation in each patient between pretreatment and after 3 months of treatment with benralizumab. The dotted line shows the mean of all patients.

The clinical parameters of the patients included in the proof-of**-**concept study are shown in Table 6. Three of the patients had NP, and three were sensitized to pollens, without present clinical relevance. All patients were in treatment with high dose inhaled corticosteroids and tiotropium bromide at entry, although no patient was treated with oral corticosteroids. Before treatment with benralizumab, *IL5RA* expression levels and eosinophil counts were high in two patients, intermediate in two, and lower in another one. After 3 months of treatment, a dramatic reduction (90–100%) of peripheral eosinophil count was observed in all patients (*P* = 0.042) (Figure 4A). *IL5RA* expression levels were reduced between 70–96% after treatment (*P* = 0.043) (Figure 4B). A strong correlation was found between *IL5RA* levels and peripheral blood eosinophil counts at pretreatment (Figure 4C) with a Pearson's correlation coefficient of 0.940 (*P* = 0.017). In addition, all patients achieved an ACT score >20 and a mean increase of 30% in FEV1, except patient 4. This patient had the highest pretreatment levels of *IL5RA* expression, and the improvement of FEV1 reached 50% (Figure 5).

**Table 6:** *IL5RA* expression levels and clinical parameters in pretreatment and after 3 months of benralizumab treatment.

	**Eosinophils/μl**	***IL5RA*, relative expression**	**FEV1, ml**	**FeNO, ppb**	**ACT**
**Patient 1**					
Pretreatment	630	1.8	1,897	43	13
3 months	10	0.6	2,462	68	22
**Patient 2**					
Pretreatment	820	29.2	1,960	-	8
3 months	10	1.2	2,520	-	24
**Patient 3^a^**					
Pretreatment	437	10.9	2,310	154	12
3 months	0	1.0	3,100	113	22
**Patient 4**					
Pretreatment	1,290	62.5	1,890	66	19
3 months	0	4.7	2,840	198	25
**Patient 5^a^**					
Pretreatment	630	12.6	2,060	65	12
3 months	10	0.8	2,630	64	21

a *Patients previously treated with other biological drugs, but no improvement was observed*.

## Discussion

Novel biological therapies have increased clinical treatment options for asthma. The necessity of identifying biomarkers to achieve a proper selection of these expensive compounds has led to the application of transcriptomic methods as a starting point for discovering new genes involved in the disease. In a previous transcriptomic study, increased expression of interleukin-4 receptor (IL-4R) on B cells was observed in allergic asthma patients (39). In the present study, we have focused on another type 2 cytokine, IL-5, which is also involved in asthma pathophysiology. Thus, IL-5RA has been analyzed due to its implication with eosinophilic-mediated immunity.

In the RNAseq study, significant gene expression differences were observed between the peripheral blood samples of controls and allergic asthmatic patients, with a top 26 differentially expressed genes, as shown in Table 3. Interestingly, most of these genes are involved in biological processes related to the immune system, suggesting their potential implication in the pathophysiology of asthma. *IL5RA* turned out to be one of the genes with the highest differential expression, which is also supported by its relevant role in asthma (16, 17) and its interactions with other immune response effector molecules that were detected in the protein-protein interaction network analysis. All proteins of the *IL5RA* cluster had already been reported to participate in functions related to inflammatory response, signal transduction and eosinophil mediated immunity, such as eosinophil chemotaxis, regulation of type 2 cytokine production or cell differentiation and proliferation (40–44). These functions are consistent with the biological processes obtained in our transcriptomic study, described in Table 4. Furthermore, differential *IL5RA* expression levels have also been described in the literature, both between asthmatic patients and controls (45, 46) and pre and post-benralizumab treatment samples (47).

Following the results obtained in the RNAseq study, we decided to carry out a qPCR validation study to evaluate the performance of the peripheral blood *IL5RA* expression levels in the diagnosis of asthma. Levels were significantly higher in asthmatic patients than in controls, independently of the type of asthma (Table 5). This result suggests that it could be a potential marker in the diagnosis of asthma. One main limitation is its correlation with eosinophil counts, as a moderate correlation was observed (*r* = 0.520). Nevertheless, as shown by green arrows in Figure 2A, there was a notable dispersion of values, and some patients had high *IL5RA* expression levels compared to their eosinophil counts and vice versa. Also, we found that patients with the same eosinophil counts can show very different *IL5RA* expression levels. For example, *IL5RA* expression values ranging from 4 to 32 were observed for counts of 200 eosinophils/μl. This distribution was also found in controls, as variability in *IL5RA* expression levels was observed at low eosinophil counts. However, the dispersion was considerably more significant in patients with high eosinophil counts. This dispersion was also observed in all subgroups according to asthma severity. It can be speculated that differences could be due to different levels of expression of the *IL5RA* by eosinophils, caused by unknown elements, such as genetic or environmental factors driving the expression of the receptor. Besides, differences could reflect the expression by other cell types, such as basophils (20). In this sense, basophils have been involved in the immunology of eosinophilic asthma (48). Furthermore, this differential expression of *IL5RA* could be related to the different responses observed to biologics directed against *IL5RA*. In fact, in the proof-of-concept study, the patient with the highest levels of *IL5RA* expression was the best responder in terms of ACT and FEV1 (see below).

Concerning the different types of asthma, *IL5RA* expression levels were elevated in both allergic and non-allergic asthma. This finding is in agreement with the fact that responses to benralizumab are not influenced by the atopy status (49). In addition, we observed a progressive increase in the expression of *IL5RA* levels from intermittent to severe asthma, which could be related to the number of eosinophils to a great extent. One streaking feature was that *IL5RA* expression levels were significantly higher in non-allergic asthma patients with NP respect to non-allergic asthma patients without NP. In this sense, it has been described that *IL5RA* expression is increased in patients with NP, particularly those with Aspirin-Exacerbated Respiratory Disease (50). Whether this could be related to response to the treatment with biologics in patients with chronic rhinosinusitis with NP remains speculative.

In the proof-of-concept pharmacogenetic study, we selected peripheral blood because it is easily accessible, a crucial characteristic of an ideal biomarker (51). In addition, as we were trying to check the *IL5RA* expression as a biomarker, we selected benralizumab treatment as it is directed against *IL5RA* (21). All patients were good responders, as demonstrated by the increase of ACT and FEV1. It is known that anti-IL-5 and anti-IL-5RA treatments do not significantly modify FeNO levels (52), as it happened in most of our patients. The increase observed in some of them could be due to acute exposure to allergens (53) because adherence to inhaled corticosteroids seemed to be appropriated. Due to the short follow-up period, exacerbations were not considered, although no patient had an exacerbation after treatment with benralizumab; even more, they almost did not require rescue bronchodilators. All patients showed a dramatic decrease in peripheral blood eosinophil counts with values between 0 and 10 eosinophils/μl, as observed in the phase III studies (21). The pretreatment levels of *IL5RA* were highly variable in patients, ranging from 1.8 to 62.5, and were strongly correlated with pretreatment peripheral blood eosinophil counts (*r* = 0.940). Thus, we believe that *IL5RA* expression level could add value to peripheral blood eosinophil counts. Accordingly, the patient with the highest *IL5RA* expression levels showed the best lung function response and reached an ACT of 25. In a very recent study, Nakajima et al. (54) described a group of super responder to benralizumab patients that had higher expression of genes related to eosinophils in peripheral blood, together with significant reductions in the expressions of genes associated with eosinophilic inflammatory responses after treatment with benralizumab, with *IL5RA* among them. So, the expression of *IL5RA* could be a useful biomarker of response, as it seems to be more discriminant than eosinophil counts.

This study is not without limitations since it is a unicentric study, and the number of patients is low. Nevertheless, this fact gives uniformity to the study. Additionally, we have mainly focused on *IL5RA*, and other genes may also be relevant in response to treatment. Nevertheless, we selected *IL5RA* by its crucial implication in the immunology of T2-asthma and because benralizumab is directed against this molecule. Finally, the proof-of-concept study has a limited number of patients and a short period of follow-up.

## Conclusion

There is an urgent need for biomarkers of response to biologics in asthma. In this study, we have explored the peripheral blood *IL5RA* expression levels as a possible useful biomarker for several reasons. First, *IL5RA* is a plausible etiopathogenic target and a biologic target for the treatment of asthma. Second, peripheral blood is easily accessible. Third, its expression is easily measurable and reproducible. Fourth, it varies in different types of asthma. And, finally, although it has a moderate correlation with eosinophils, *IL5RA* expression levels probably do not reflect the same, as these levels vary for a particular count of peripheral blood eosinophils. Further studies are required for confirming the findings of the present study.

## Data Availability Statement

The required data is now publicly accessible in the NCBI repository with the code PRJNA686899.

## Ethics Statement

The studies involving human participants were reviewed and approved by The Clinical Research Ethics Committee of the Institute for Biomedical Research of Salamanca (IBSAL) (PI 2020-02-433). The patients/participants provided their written informed consent to participate in this study.

## Author Contributions

SE-P, DH-J, AG-S, ME, MM, JR-G, JT, MI-G, CS, and ID have contributed in designing research studies, conducting experiments, acquiring data, analyzing data, and writing the manuscript. All authors read and approved the final manuscript.

## Conflict of Interest

ID declares having received honoraria for participation in speakers' bureaus or advisory boards from Astra-Zeneca, GSK, and Sanofi. The remaining authors declare that the research was conducted in the absence of any commercial or financial relationships that could be construed as a potential conflict of interest.
